# Deutschlandweite Evaluation der ärztlichen Weiterbildung in Psychiatrie und Psychotherapie

**DOI:** 10.1007/s00115-024-01796-1

**Published:** 2025-01-27

**Authors:** Nina Schubotz, Julia-Maleen Kronsbein, Angela Zapp, Rebecca Reichel

**Affiliations:** 1 Alexianer St. Joseph-Krankenhaus Berlin, Gartenstraße 1, 13088 Berlin, Deutschland; 2 Praxis für Psychiatrie und Psychotherapie Usedom, Klenzestraße 14, 17424 Heringsdorf, Deutschland; 3 Zentralinstitut für Seelische Gesundheit (ZI), J5, 68159 Mannheim, Deutschland; 4 Universitätsklinik für Psychiatrie und Psychotherapie Tübingen, Calwerstraße 14, 72076 Tübingen, Deutschland

**Keywords:** Facharztweiterbildung, Sorgearbeit, Qualitätssicherung, Nachwuchsmangel, Arbeitsbedingungen, Specialist training, Care work, Quality assurance, Lack of young talent, Working conditions

## Abstract

**Hintergrund:**

Bislang existiert keine Evaluation der Weiterbildungs- und Arbeitsbedingungen in der Facharztweiterbildung Psychiatrie und Psychotherapie in Deutschland. Um dem Nachwuchsmangel entgegenzuwirken und die Versorgung der Patient:innen langfristig sicherzustellen, ist eine Qualitätssicherung unabdingbar.

**Ziel der Arbeit:**

Ziel der Evaluation ist es, Stärken und Schwächen der Weiterbildung zu identifizieren und Maßnahmen zur Verbesserung abzuleiten. Für eine bessere Einordnung wird außerdem ein Vergleich mit anderen Facharztweiterbildungen vorgenommen.

**Material und Methoden:**

Die onlinebasierte Befragung erfolgte vom 15.05.2023 bis zum 30.06.2023. Die Ergebnisse beziehen sich auf 315 Fragebögen von 202 Ärzt:innen in Weiterbildung und 113 Fachärzt:innen (Facharzttitel seit weniger als 3 Jahren).

**Ergebnisse:**

Insgesamt 64 % der Befragten sind mit Qualität und Umsetzung des allgemeinen Psychiatrie-Teils zufrieden, nur 11 % erwägen aufgrund der Weiterbildungsbedingungen einen Wechsel der Fachrichtung und 18 % eine Verlegung ihres Arbeitsplatzes ins Ausland. Verbesserungsbedarf zeigt sich bei der Einarbeitung sowie bei der Organisation der Weiterbildung. Zudem werden ein erheblicher Zeit- und Kostenaufwand für verpflichtende externe Weiterbildungsveranstaltungen und eine mangelnde Vereinbarkeit der Weiterbildung mit Forschung, Lehre und Care-Arbeit beanstandet.

**Diskussion:**

Insgesamt zeigt sich, dass die Arbeitsbedingungen besser sind als in anderen Fächern. Hinsichtlich des Verbesserungspotenzials ist die Schaffung einer Finanzierung der Weiterbildung unerlässlich, zudem können Zertifikate für gute Weiterbildung sowie die Veröffentlichung von Positivbeispielen zur Qualitätssicherung und -verbesserung beitragen.

**Zusatzmaterial online:**

Zusätzliche Informationen sind in der Online-Version dieses Artikels (10.1007/s00115-024-01796-1) enthalten.

## Abstract (en)

**Background:**

To date there has been no evaluation of further training and working conditions in medical specialist training in psychiatry and psychotherapy in Germany. In order to counteract the lack of young talent and ensure long-term care for patients, quality assurance is essential.

**Aim of the work:**

The evaluation aims to identify strengths and weaknesses of the further training and to derive measures for improvement. For a better classification, a comparison is also made with other disciplines with medical specialist further training.

**Material and methods:**

The online-based survey took place from 15 May 2023 to 30 June 2023. The results refer to 315 questionnaires from 202 physicians in further training and 113 medical specialists (specialist title for less than 3 years).

**Results:**

Of those surveyed 64% were satisfied with the quality and implementation of the general psychiatry part, only 11% were considering changing their specialty and 18% were considering moving their job abroad due to the training conditions. There is a need for improvement in induction and in the organization of the training. In addition, significant time and financial burdens for mandatory external training events as well as the lack of compatibility between training and research, teaching and care work responsibilities were criticized.

**Discussion:**

Overall, it appears that the working conditions are better than in other disciplines. With respect to the potential for improvement, the creation of financing for further training is essential; certificates for high quality training and the publication of positive examples can also contribute to quality assurance and improvement.

## Hintergrund und Fragestellung

Die Facharztweiterbildung ist der Karrierebeginn junger Ärzt:innen und der Beginn ihrer klinischen Tätigkeit. Die erforderlichen Weiterbildungsinhalte, -zeiten und -kompetenzen werden durch die Weiterbildungsordnung der jeweiligen Landesärztekammer vorgegeben sowie durch diese überprüft. Die (Muster‑)Weiterbildungsordnung der Bundesärztekammer ist dabei nicht bindend, sodass sich die Weiterbildungsordnung der einzelnen Landesärztekammern unterscheiden kann. Die ärztliche Weiterbildung können nur durch die Landesärztekammern ermächtigte Einrichtungen anbieten. Eine direkte Finanzierung der Facharztweiterbildung existiert bislang nicht.

Der ärztliche Nachwuchsmangel betrifft alle Fächer [12]. In der Psychiatrie und Psychotherapie ist die fortbestehende Stigmatisierung psychischer Erkrankungen und psychiatrischer Institutionen eine zusätzliche Hürde bei der Nachwuchsgewinnung. Eine qualitativ hochwertige Weiterbildung und Zufriedenheit der Ärzt:innen mit der Weiterbildung hat einen wichtigen Stellenwert für die Sicherstellung der zukünftigen Patient:innenversorgung. Eine regelmäßige Evaluation der Weiterbildung bildet die Grundlage für strukturelle Verbesserungen und stellt ein wichtiges Qualitätsmerkmal dar. In den letzten Jahren wurden viele Facharztweiterbildungen evaluiert [1, 3, 8, 9, 12, 14–16]. Eine Evaluation der psychiatrischen Facharztweiterbildung stand bislang aus. Es erfolgte eine onlinebasierte Befragung von Ärzt:innen an deutschen Weiterbildungseinrichtungen über Surveymonkey® (Survey Monkey Inc., San Mateo, CA, USA) im Zeitraum vom 15.05.2023 bis zum 30.06.2023. Die Befragung wurde durch die Nachwuchsinitiative Generation PSY der Deutschen Gesellschaft für Psychiatrie und Psychotherapie, Psychosomatik und Nervenheilkunde e. V. (DGPPN) entwickelt und durchgeführt.

## Studiendesign und Untersuchungsmethoden

Zielgruppe der Befragung waren alle Ärzt:innen in Facharztweiterbildung Psychiatrie und Psychotherapie sowie Fachärzt:innen, deren Facharztprüfung zum Zeitpunkt der Befragung weniger als drei Jahre zurücklag. Der Aufruf zur Teilnahme erfolgt über die folgenden Wege: Die Mitglieder der DGPPN sowie des Berufsverbands Deutscher Psychiater (BVDP) wurden über die Mitglieder-Newsletter informiert. Dabei wurde sowohl die Zielgruppe direkt angesprochen als auch Weiterbildungsermächtigte zur Weiterleitung an die Zielgruppe aufgerufen. Zudem erfolgte die Bekanntmachung über die Lehrstuhlinhaber für Psychiatrie & Psychotherapie e. V. (LIPPS), den Arbeitskreis der Chefärztinnen der Kliniken für Psychiatrie & Psychotherapie an Allgemeinkrankenhäusern (ackpa), die Bundesvereinigung leitender Ärzte und Ärztinnen der Kliniken für Psychiatrie und Psychotherapie e. V. (Bundesdirektorenkonferenz/BDK) und das Bündnis Junge Ärztinnen und Ärzte (BJÄ). Weiterhin erfolgte eine Verbreitung über Blogposts (Psychiatrie to go) und Instagram (Operation Karriere des Deutschen Ärzteverlags) und ein direkter Aufruf an alle ambulanten Weiterbildungsermächtigten mit digitalen Kontaktdaten auf der Internetseite der jeweiligen Landesärztekammer.

Der Fragebogen umfasste 64 Fragen zu 8 Themenfeldern:

1. Basisdaten (10 Fragen)
2. Gesamturteil/Konsequenzen aus Weiterbildungs- und Arbeitsbedingungen (7 Fragen)
3. Arbeitsbedingungen (8 Fragen)
4. Weiterbildungskonzept/Rotationen (12 Fragen)
5. Interne Weiterbildungsveranstaltungen (5 Fragen)
6. Externe Weiterbildungsveranstaltungen (6 Fragen)
7. Einarbeitung/Anleitung/Supervision (7 Fragen)
8. Vereinbarkeit der Weiterbildung mit Care-Arbeit/Privatinteressen, Lehre und Forschung (9 Fragen)

Abhängig von der Antwortlogik mussten die Befragten minimal 56 und maximal 64 Fragen beantworten. Die Auswertung erfolgte mit SPSS Statistics (IBM, New York, USA, Version 29.0.0.0).

## Ergebnisse

**Abb. 1:**
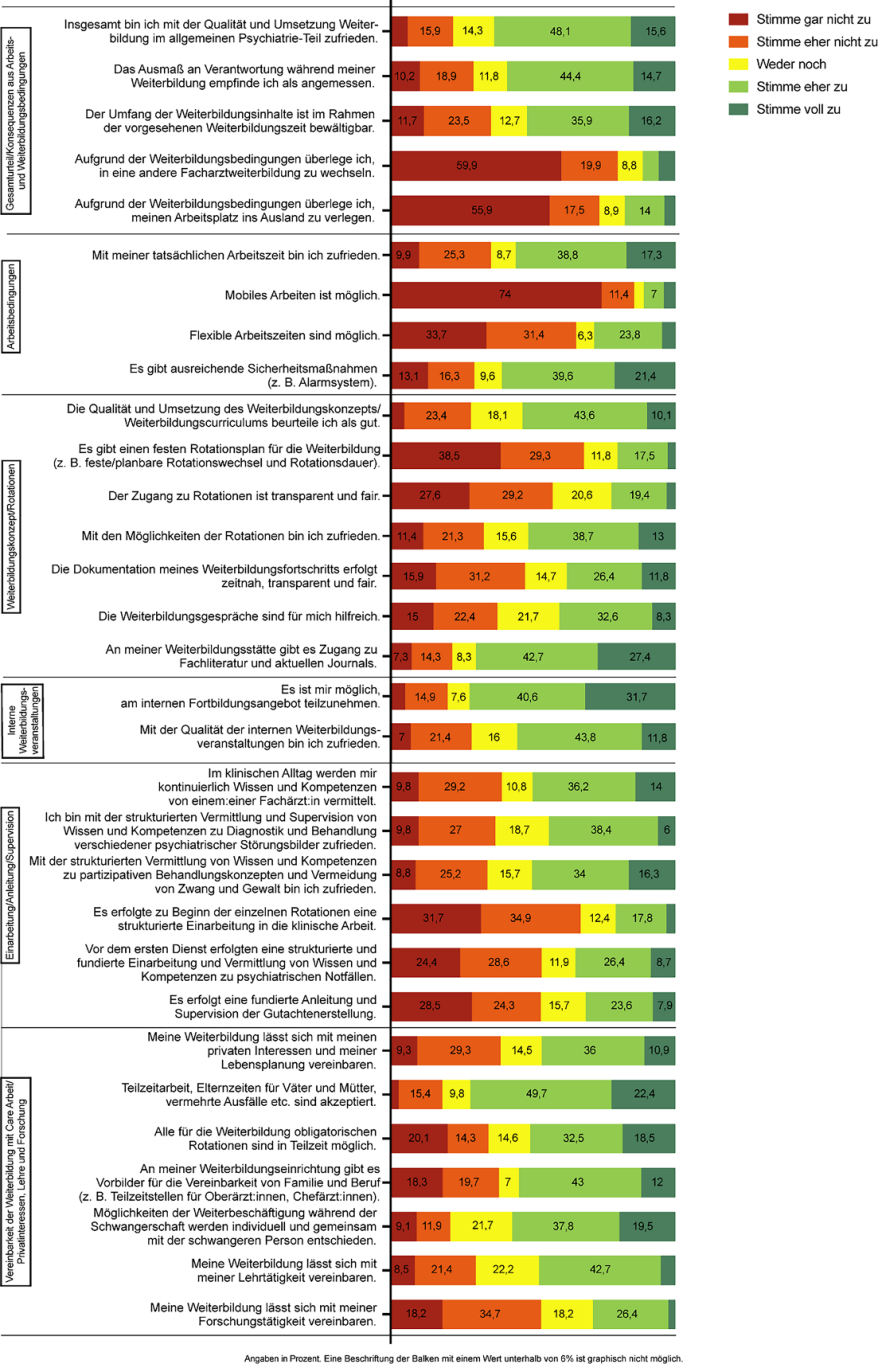
Ergebnisse der Likert-Skalen in den einzelnen Themenbereichen II

Es wurden insgesamt 315 Fragebögen ausgewertet. Die Stichprobe bestand aus 202 (64 %) Ärzt:innen in Weiterbildung und 113 (36 %) Fachärzt:innen zwischen 23 und 63 Jahren (Durchschnittsalter 36,9 Jahre, SD 7,3). Weitere Charakteristika der Gesamtstichprobe können eTabelle 1 im elektronischen Anhang entnommen werden. Die Ergebnisse der Likert-Skalen sind Abb. 1 zu entnehmen*.*

Ende 2023 waren laut Erhebung der Bundesärztekammer von insgesamt 12.957 im Fach Psychiatrie und Psychotherapie tätigen Ärzt:innen 1263 jünger als 40 Jahre (9,7 %). Stationär tätig waren dabei 6533 Ärzt:innen, davon 998 jünger als 40 Jahre (15,3 %; [17]). Eine Differenzierung in Ärzt:innen in Weiterbildung und Facharzt:innen erfolgt in der Statistik der Bundesärztekammer nicht, sodass die genaue Gesamtzahl der Zielgruppe der Ärzt:innen in Weiterbildung nicht eruierbar ist. Folglich kann auch der exakte Anteil der Befragten unserer Umfrage an der Gesamtkohorte nicht bestimmt werden. Unter der Annahme, dass die überwiegende Mehrheit der 998 stationär tätigen Ärzt:innen unter 40 Jahre sich in Weiterbildung befindet oder die Facharztprüfung erst kürzlich absolviert hat, basieren die Ergebnisse unserer Umfrage auf Aussagen von ca. 32 % dieser Grundgesamtheit.

### Gesamturteil

Insgesamt 64 % der Befragten sind mit der Qualität und Umsetzung des allgemeinen Psychiatrie-Teils zufrieden. Das Ausmaß der Verantwortung während der Weiterbildung empfinden 59 % als angemessen. 52 % erachten den Umfang der Weiterbildungsinhalte im Rahmen der vorgesehenen Weiterbildungszeit als bewältigbar. Nur 11 % erwägen, aufgrund der Weiterbildungsbedingungen in eine andere Facharztweiterbildung zu wechseln, und 18 % ziehen deshalb in Erwägung, ihren Arbeitsplatz ins Ausland zu verlegen.

### Arbeitsbedingungen

Mit ihrer Arbeitszeit sind 56 % der Befragten zufrieden. Detaillierte Informationen bezüglich der Arbeitszeit können Tab. 1 entnommen werden. Nur bei 11 % ist mobiles Arbeiten in der Weiterbildungseinrichtung möglich, bei 29 % bestehen flexible Arbeitszeiten. Die Sicherheitsmaßnahmen empfinden 61 % als ausreichend.

**Tab. 1:** Arbeitsbedingungen

Vertragliche Arbeitszeit	62,5 % Vollzeit (mind. 38 h/Woche), 37,5 % Teilzeit
Tatsächliche Arbeitszeit	79,6 % Überstunden, durchschnittlich 5,7 Überstunden pro Woche (SD 4,3)
Dienstarten	78 % Nachtdienste, 80 % Tagdienste am Wochenende, 60 % Tagdienste außerhalb des Wochenendes, 54 % Zwischendienste, 71 % 24-h-Dienste
Dienstbelastung pro Monat	4,5 Dienste pro Monat (SD 2,7)
	2,7 Dienste äquivalent zu 24-h-Dienst pro Monat (SD 2,1) [Nachtdienst/Tagdienste = Faktor 0,5; 24-h-Dienst = Faktor 1; Zwischendienst = Faktor 0,25]

### Weiterbildungskonzept und Rotationen

Ein strukturiertes Weiterbildungskonzept existiert bei 60 % der Befragten. Mit der Qualität und Umsetzung des Weiterbildungskonzepts sind 54 % zufrieden. Bei 70 % existiert ein Zugang zu Fachliteratur und aktuellen Journals. Bei 68 % sind die Rotationszeitpunkte und die jeweilige Rotationsdauer nicht festgelegt oder geplant. Auch einen fairen und transparenten Zugang zu den einzelnen Rotationen sehen 57 % nicht gegeben. Die Dokumentation des Weiterbildungsfortschritts erfolgt nur bei 38 % zeitnah, transparent und fair. Weiterbildungsgespräche finden bei 72 % jährlich oder häufiger statt. Jedoch empfinden nur 41 % diese Gespräche als hilfreich.

### Interne Weiterbildungsveranstaltungen

Bei 96 % finden regelmäßig interne Fortbildungen zu weiterbildungsrelevanten Themen statt, bei 64 % wöchentlich. 72 % der Befragten ist es möglich, am internen Fortbildungsangebot teilzunehmen. 56 % der Befragten sind mit der Qualität der internen Weiterbildungsveranstaltungen zufrieden oder eher zufrieden.

### Einarbeitung, Anleitung und Supervision

Ein Gespräch zur Planung der Weiterbildung innerhalb der ersten Monate der Anstellung findet bei 49 % statt. Nur 31 % der Befragten erhalten eine ausreichende Einarbeitung in die klinische Arbeit zu Beginn der einzelnen Rotationen. 99 % der Befragten machen Bereitschaftsdienste. Vor dem ersten Dienst erhalten jedoch nur 35 % eine strukturierte und fundierte Einarbeitung. 50 % bekommen im klinischen Alltag kontinuierlich Wissen und Kompetenzen von einem Facharzt oder einer Fachärztin vermittelt. Mit der strukturierten Vermittlung und Supervision von Wissen und Kompetenzen zu Diagnostik und Behandlung verschiedener psychiatrischer Störungsbilder sind 44 % zufrieden, mit der Wissens- und Kompetenzvermittung zu partizipativen Behandlungskonzepten und Vermeidung von Zwang und Gewalt 50 %. Die Anleitung und Supervision der Gutachtenerstellung erachten 53 % als nicht ausreichend fundiert.

### Vereinbarkeit der Weiterbildung mit Care-Arbeit und Privatinteressen

Insgesamt 47 % der Befragten sind mit der Vereinbarkeit der Weiterbildung und ihrer Privatinteressen zufrieden. 46 % tragen Verantwortung für ein oder mehrere Kinder. Die Akzeptanz für Teilzeitarbeit, Elternzeiten und vermehrte Ausfälle sehen 72 % der Befragten mit Kindern gegeben. 51 % der Befragten werden Teilzeitoptionen für alle obligatorischen Rotationen ermöglicht. Vorbilder für die Vereinbarkeit von Familie und Beruf haben 55 % an ihrer Weiterbildungseinrichtung. Eine individuelle und gemeinsame Entscheidung bezüglich der Weiterbeschäftigung während der Schwangerschaft gibt es bei 57 %.

### Vereinbarkeit der Weiterbildung mit Forschung und Lehre

Insgesamt 37 % der Befragten sind in der Lehre und 38 % in der Forschung tätig. Ihre Lehrtätigkeit sehen 48 % der Lehrenden mit der Weiterbildung gut vereinbar. Einer guten Vereinbarkeit von Forschung und Weiterbildung stimmen jedoch nur 29 % der in der Forschung tätigen zu.

### Externe Weiterbildungsveranstaltungen

Nur 11 % können alle verpflichtenden Veranstaltungen in ihrer Weiterbildungseinrichtung absolvieren. Die Psychotherapiestunden können von 60 % vollständig und von 25 % teilweise an der Weiterbildungseinrichtung absolviert werden. 71 % der Befragten müssen verpflichtende Weiterbildungsanteile selbst finanzieren (vgl. eTabelle 2 im elektronischen Anhang). Bei 84 % werden Zeiten für Weiterbildungsveranstaltungen außerhalb der regulären Arbeitszeit nicht vergütet. 56 % wenden bis zu 5 h, weitere 39 % 5–10 h pro Woche außerhalb der regulären Arbeitszeit für externe Weiterbildungsinhalte sowie Vor- und Nachbereitung auf. 16 % erhalten einen pauschalen Weiterbildungszuschuss ihres Arbeitgebers. Die Höhe des Zuschusses liegt bei 78 % davon bei bis zu 1000 € pro Jahr.

## Diskussion

Die Befragung zeigt, dass die Arbeitsbedingungen im Fachgebiet Psychiatrie und Psychotherapie besser sind als im fächerübergreifenden Vergleich [11]. Im Vergleich zur durchschnittlichen Arbeitszeit von Ärzt:innen in Weiterbildung in der fächerübergreifenden Befragung des Hartmannbunds von 2021 [11] zeigt sich eine deutlich geringere tatsächliche Arbeitszeit in der Psychiatrie und Psychotherapie. Auch eine Teilzeittätigkeit wird in der Psychiatrie und Psychotherapie häufiger in Anspruch genommen. Es ist denkbar, dass der Aufwand für externe Weiterbildungsveranstaltungen ebenfalls ein Grund für eine Teilzeittätigkeit sein könnte. Es sollten daher mögliche strukturelle Gründe für eine Teilzeittätigkeit genauer untersucht werden.

Es zeigt sich ein Verbesserungsbedarf in den Bereichen Einarbeitung, Supervision und Weiterbildungsplanung. Besonders die Einarbeitung in psychiatrische Notfälle ist sicherheitsrelevant und daher von zentraler Bedeutung. Flächendeckend sollten zu Beginn der Weiterbildung praxisorientierte Seminare zur Notfallpsychiatrie angeboten werden. Darüber hinaus spielt die Organisation des Rotationssystems eine wesentliche Rolle, wobei ein frühzeitiger Einsatz in der Akut- und Allgemeinpsychiatrie entscheidend ist. Insbesondere in psychiatrischen Fachkliniken ist zudem die Einarbeitung in die Vorgehensweisen bei somatischen Notfällen bedeutsam.

Bezüglich der Weiterbildungsplanung können Leitfäden für die Weiterbildungsgespräche hilfreich sein. Zudem sollten alle Weiterbildungseinrichtungen den Ärzt:innen in Weiterbildung das Weiterbildungskonzept, einschließlich der Umsetzung externer Weiterbildungsinhalte, zu Beginn der Weiterbildung transparent und schriftlich vorlegen. Die Einführung standardisierter Rotationsleitfäden in Kooperation mit den Fachgesellschaften könnte die Situation verbessern. Darüber hinaus kann die Zuweisung von Mentor:innen für die gesamte Weiterbildungszeit sowie ein Peer-Mentoring innerhalb der Ärzt:innen in Weiterbildung die Einarbeitung und individuelle Weiterbildungsplanung erheblich erleichtern. Um die Umsetzbarkeit und Effektivität solcher Maßnahmen zu evaluieren, könnten diese an ausgewählten Kliniken durch Pilotprojekte erprobt und weiterentwickelt werden.

Bei der Vergabe der Rotationen wird ein Mangel an Transparenz, Fairness und Planbarkeit deutlich. Der Einsatz der Ärzt:innen in Weiterbildung in unterschiedlichen Bereichen zum Zwecke ihres persönlichen Wissens- und Kompetenzaufbaus steht im Spannungsfeld zur Behandlungskontinuität der Patient:innen und der Förderung langfristiger Teambindungen. Die Weiterbildungsordnung definiert jedoch klar Wissens- und Kompetenzerwerb in verschiedenen Bereichen. Aus diesem Grund ist es unerlässlich, dass Weiterbildungseinrichtungen mit voller Weiterbildungsermächtigung diese Rotationen innerhalb der psychiatrischen Weiterbildungszeit und in Teilzeit ermöglichen.

Die Befragung zeigt, dass flexible Arbeitszeiten und mobiles Arbeiten während der Weiterbildung kaum möglich sind. Gleichzeitig fällt in der klinischen Tätigkeit ein erheblicher Dokumentationsaufwand an, wie etwa das Verfassen von Arztbriefen, die Dokumentation von Visiten und Gesprächen, Anordnungen, Gutachten sowie Stellungnahmen an Gerichte. Bei vollständiger Digitalisierung könnten viele dieser Aufgaben ortsunabhängig und mit größerer zeitlicher Flexibilität erledigt werden. Die Befragung des Hartmannbundes verdeutlicht, dass Ärzt:innen in Weiterbildung die Digitalisierung als wichtig erachten, effizient digitalisierte Arbeitsplätze jedoch rar sind [7]. Flexible Arbeitszeiten und mobiles Arbeiten beeinflussen die Vereinbarkeit von beruflicher Tätigkeit und Care-Arbeit erheblich, insbesondere da die Weiterbildungszeit häufig in die Phase der Familiengründung fällt. Daher sollten diese Arbeitsmodelle in der Psychiatrie stärker gefördert und ausgebaut werden.

Die Gesamtheit der Befragten beurteilt die Vereinbarkeit der Weiterbildung mit ihren privaten Interessen unterschiedlich, 50 % sind zufrieden und 39 % unzufrieden. Während die Vereinbarkeit von Care-Arbeit und Weiterbildung an einigen Weiterbildungseinrichtungen schon gut gelingt, erfahren Eltern an anderen Weiterbildungseinrichtungen noch wenig Akzeptanz und strukturelle Unterstützung. Dieses Ergebnis deckt sich mit den Evaluationen anderer Facharztweiterbildungen [8, 14]. Die Einführung transparenter Zertifikate, die Weiterbildungseinrichtungen hinsichtlich ihrer Vereinbarkeit von Beruf und Familie bewerten, könnte hier Anreize schaffen, die Bedingungen zu verbessern.

In Anlehnung an die vom Deutschen Ärztinnenbund (DÄB) vorgestellten Positivbeispiele zum Mutterschutz für schwangere Ärztinnen [2] könnte die Bekanntmachung von Positivbeispielen zu Maßnahmen für eine bessere Vereinbarkeit von Care-Arbeit und Arztberuf im psychiatrischen Versorgungssystem und speziell während der Facharztweiterbildung dazu beitragen, die Arbeitsbedingungen für Ärzt:innen mit Verantwortung für Kinder oder zu pflegende Angehörige zu verbessern. Dies würde nicht nur die Attraktivität der Weiterbildung, sondern auch des Fachs Psychiatrie insgesamt erhöhen.

Verbesserungsbedarf zeigt sich bei der Vereinbarkeit der Weiterbildung mit einer Forschungstätigkeit. Um dem Nachwuchsmangel, insbesondere an Universitätskliniken, entgegenzuwirken und auch künftig das einzigartige Potenzial von Ärzt:innen, die sowohl in der Patientenversorgung als auch in der Forschung tätig sind, zu nutzen, müssen die Bedingungen für forschende Ärzt:innen in Weiterbildung flächendeckend verbessert werden. Zentrale Maßnahmen sind eine verbindliche Anrechenbarkeit der Forschungstätigkeit auf die Facharztweiterbildung, eine äquivalente Bezahlung forschender und klinisch tätiger Ärzt:innen sowie die strukturierte Vermittlung von Forschungskompetenzen.

Kritik deckt die Befragung bei den Rahmenbedingungen der Weiterbildung auf. Insbesondere wird die vielerorts notwendige Inanspruchnahme externer Weiterbildungsveranstaltungen beanstandet, die mit unbezahlten Weiterbildungszeiten und privat zu tragenden Kosten verbunden sind. Die externen Veranstaltungen sind insbesondere, wenn auch nicht ausschließlich, für Weiterbildungsinhalte aus dem speziellen Psychotherapie-Teil erforderlich. Die Selbsterfahrung sollte, um ihren Zweck erfüllen zu können und Machtmissbrauch zu verhindern, extern absolviert und durch von der weiterbildungsermächtigten Einrichtung unabhängige und qualifizierte Psychotherapeut:innen erfolgen. Da Selbsterfahrung nicht in die klinische Arbeit integriert werden kann, stellt sie im Vergleich zu externen Pflichtveranstaltungen anderer Facharztweiterbildungen eine besondere Herausforderung dar.

Die Organisation und Umsetzung der in die Facharztweiterbildung integrierten Psychotherapieweiterbildung bedarf dringender Verbesserungen. Es müssen Finanzierungsmöglichkeiten geschaffen und realistische Konzepte entwickelt werden, die eine qualitativ hochwertige Psychotherapieweiterbildung innerhalb der vorgesehenen Weiterbildungszeit flächendeckend ermöglichen. Regionale Weiterbildungsverbünde mehrerer Kliniken, möglicherweise unter Einbeziehung bestehender Psychotherapieweiterbildungsinstitute, könnten hierbei eine sinnvolle Lösung sein. Privat anfallende Kosten sollten durch Zuschüsse oder die vollständige Kostenübernahme durch die Weiterbildungseinrichtung kompensiert werden. Die Psychotherapieweiterbildung sollte während der regulären Arbeitszeit oder im Rahmen voll entlohnter Fortbildungstage erfolgen. Alternativ sollte eine Kompensation der Weiterbildungszeiten durch Überstundenregelungen sichergestellt werden.

Bislang mangelt es für alle Facharztweiterbildungen an einer direkten Finanzierung. Das Bündnis Junge Ärztinnen und Ärzte fordert in seinem Positionspapier 2023 klar die Notwendigkeit der Schaffung einer flächendeckenden Finanzierung für alle Facharztweiterbildungen und sieht diesbezüglich eine Chance in der Krankenhausreform, die Finanzierung der Weiterbildung einzuplanen und mitzudenken [10].

Für die Facharztweiterbildung sind die jeweiligen Landesärztekammern zuständig. Trotz erheblicher struktureller und finanzieller Unterschiede zwischen den einzelnen Weiterbildungseinrichtungen hängt die Qualität der Weiterbildung auch vom Vorhandensein und der Qualität eines Weiterbildungscurriculums/-konzepts sowie von der Qualifikation der an der Weiterbildung beteiligten Personen ab. Zur Qualitätssicherung können Zertifikate beitragen, beispielsweise die DGPPN-Zertifizierung [7]. Das Zertifizierungsverfahren der DGPPN für Weiterbildungseinrichtungen basiert auf den von der europäischen Arztfachgesellschaft (European Union of Medical Specialists, UEMS) erarbeiteten Kriterien zur Qualitätssicherung und -optimierung und beinhaltet eine zweitägige Visitation vor Ort. Zertifizierungen bieten einen Anreiz für Weiterbildungseinrichtungen und schaffen Transparenz für Ärzt:innen in Weiterbildung, Berufseinsteiger:innen und interessierte Studierende.

Zudem sollte eine regelmäßige Qualitätssicherung der Weiterbildung durch die einzelnen Landesärztekammern erfolgen. Die Abgeordneten des 126. Deutschen Ärztetages 2022 haben den Vorstand der Bundesärztekammer aufgefordert, auf eine bundeseinheitliche, regelmäßige, anonymisierte Evaluation der Weiterbildung und stetige Verbesserung der Weiterbildung hinzuwirken [4].

## Ausblick

Die Weiterbildungsevaluation zeigt Schwächen und Stärken der aktuellen Facharztweiterbildung auf. Um Änderungen der Zufriedenheit zu erfassen, insbesondere vor dem Hintergrund der zwischen 2020 und 2022 in Kraft getretenen neuen Weiterbildungsordnung, sollten zukünftig regelmäßige Befragungen erfolgen. Zudem ist eine genauere Analyse der relevanten Schwachstellen erforderlich. Beispielsweise sollten die Hintergründe der bislang nur sehr eingeschränkten Möglichkeiten des mobilen Arbeitens und flexible Arbeitszeiten in einer weiteren Evaluation detaillierter untersucht sowie die aktuellen Hürden und die von Ärzt:innen in Weiterbildung und leitenden Ärzt:innen wahrgenommenen Vor- und Nachteile erfasst werden. Auch hinsichtlich der noch deutlich verbesserungsbedürftigen Vereinbarkeit von Weiterbildung und Forschung regen wir eine genauere Exploration der aktuellen Situation an universitären Weiterbildungseinrichtung an, um daraus konkrete Maßnahmen zur Verbesserung ableiten zu können. In Pilotprojekten könnten einzelne Aspekte – wie der Ausbau mobilen Arbeitens, die Flexibilisierung der Arbeitszeiten, die Einführung von Kursen zu psychiatrischen Notfällen und die Einarbeitung in die Diensttätigkeit neuer Mitarbeiter:innen – an Einrichtungen mit unterschiedlichen strukturellen Rahmenbedingungen für einen festgelegten Zeitraum eingeführt und evaluiert werden. Auch die Weiterbildungsbedingungen in ambulanten Einrichtungen sollten genauer exploriert werden. Hierbei ist eine enge Kooperation mit der Fachgesellschaft (DGPPN) sowie dem Berufsverband (BVDP) sinnvoll, um die im fächerübergreifenden Vergleich guten Arbeitsbedingungen in Psychiatrie und Psychotherapie weiter fortzuentwickeln.

## Fazit für die Praxis

- Es sollte eine Finanzierung der Weiterbildung zur Qualitätssicherung und Ermöglichung einer kostenfreien Weiterbildung innerhalb der Arbeitszeit geschaffen werden.
- Die jährlichen Weiterbildungsgespräche sollten zur Planung der Weiterbildung, insbesondere der obligatorischen externen Weiterbildungsanteile genutzt werden.
- Es sollten Strukturen zur Einarbeitung, insbesondere in Bereitschaftsdienste und psychiatrische Notfälle, in allen Weiterbildungseinrichtungen geschaffen werden.
- Die regelmäßige Anleitung und Supervision im klinischen Alltag, im Umgang mit Zwang und Gewalt und bei der Gutachtenerstellung müssen sichergestellt werden.
- Die Vereinbarkeit der Weiterbildung mit einer Forschungstätigkeit sollte strukturell verbessert werden.
- Mobiles Arbeiten sollte ausgeweitet und flexible Arbeitszeitmodelle etabliert werden.
- Positivbeispiele für eine gelungene Vereinbarkeit von Weiterbildung und Care-Arbeit zur flächendeckenden Schaffung guter Rahmenbedingungen für Ärzt:innen mit Verantwortung für Kinder und zu pflegende Angehörige sollten veröffentlicht werden.

## Supplementary Information

eTabelle 1: Charakteristika der Gesamtstichprobe

eTabelle 2: Externe Weiterbildungsveranstaltungen/-inhalte

## Data Availability

Die Originaldaten können bei der Korrespondenzautorin angefragt werden.
