# Supplementary material for: Nationwide evaluation of medical training in psychiatry and psychotherapy in Germany
Source: Nervenarzt. 2025 Jan 27;97(1):60–6. [Article in German] doi: 10.1007/s00115-024-01796-1 (PMC12808157; doi:10.1007/s00115-024-01796-1)
Supplement: Supplementary file 1 — eTabelle 1: Charakteristika der Gesamtstichprobe [file 115_2024_1796_MOESM1_ESM.docx]

**eTabelle 1:** Charakteristika der Gesamtstichprobe

| Charakteristika der Gesamtstichprobe |
| --- |
| Gesamt (315): 202 (64%) Ärzt:innen in Weiterbildung, 113 (36%) Fachärzt:innen  Geschlecht: m 35,2%, w 64,1 %, d 0,6%  Alter: 36,9 Jahre (min. 23, max. 63, SD=7,3)  Weiterbildungsstand: Ärztinnen in Weiterbildung: 1.J. (12%), 2.J. (13%), 3. J. (14%), 4. J. (13%), 5. J.  (30%), ab 6. J. (18%), Fachärzt:innen:seit 1 J. (58%), 2 J.  (31%), 3 J. (12%)  Bundesland: Baden-Württemberg 13%, Bayern 17%, Berlin 15%, Brandenburg 5%, Bremen 1%, Hamburg 3%, Hessen 7%, Mecklenburg-Vorpommern 0,6%, Niedersachsen 5%, Nordrhein-Westfalen 18%, Rheinland-Pfalz 4%, Saarland 0,3%, Sachsen-Anhalt 3%, Sachsen 3%, Schleswig-Holstein 3%, Thüringen 3%)  Tätigkeitsort: Großstadt 65%, Kleinstadt 27%, 8% ländliche Region  Versorgungsauftrag:  88,2% stationär mit Versorgungsauftrag, 8,7% stationär ohne Versorgungsauftrag, 3,2% ambulant  Art der Weiterbildungseinrichtung: Fachklinik 40%, Universitätsklinik 31%, Psychiatrie im Allgemeinkrankenhaus 21%, Forensische Klinik 2%, Psychiatrische Praxis 2%, Nervenärztliche Praxis 2%, (Sonstiges: u.a. Suchtspezifische Rehaklinik, Berufliche Rehaklinik, Tagesklinik, Medizinischer Dienst) 2%  Therapierichtung: 74% Verhaltenstherapie, 25% tiefenpsychologisch-fundierte Psychotherapie, 0,3% analytische Psychotherapie, 0,7% systemische Therapie |
