# Supplementary material for: Nationwide evaluation of medical training in psychiatry and psychotherapy in Germany
Source: Nervenarzt. 2025 Jan 27;97(1):60–6. [Article in German] doi: 10.1007/s00115-024-01796-1 (PMC12808157; doi:10.1007/s00115-024-01796-1)
Supplement: Supplementary file 2 — eTabelle 2: Externe Weiterbildungsveranstaltungen/-inhalte [file 115_2024_1796_MOESM2_ESM.docx]

**eTabelle 2:** Externe Weiterbildungsveranstaltungen/ -inhalte

| Externe Weiterbildungsveranstaltungen (verpflichtend) | extern | selbst finanziert |
| --- | --- | --- |
| Theoriestunden | 42% | 30% |
| Supervision Psychotherapiestunden | 34% | 31% |
| Balintgruppe/ Fallarbeit | 46% | 35% |
| Entspannungsverfahren | 53% | 36% |
| Selbsterfahrung | 83% | 61% |
| Zweitverfahren Psychotherapie | 29% | 20% |
| Forensische Psychiatrie | 19% |  |
| Kriseninterventionsseminar | 5,2% | 7,2% |
| Fremdrater-Seminar | 20,9% | 10,8% |
| Sozialpsychiatrie | 8,8% |  |
| Konsil- und Liaisondienst | 12% | 6,9% |
| Psychopharmakologie und somatische Behandlungsverfahren | 11% |  |
